# Ethical design as a prerequisite for translational microbiome science

**DOI:** 10.1186/s40168-026-02371-3

**Published:** 2026-04-07

**Authors:** Jo-Ann S Passmore, Abigail Nieves Delgado, Anna-Ursula Happel

**Affiliations:** 1 Centre for Epidemic Response and Innovation (CERI), Stellenbosch University, Tygerberg, South Africa; 2 Department of Pathology, Faculty of Health Sciences, University of Cape Town, Cape Town, South Africa; 3 Centre for the AIDS Programme of Research in South Africa (CAPRISA), Durban, South Africa; 4 Freudenthal Institute, Utrecht University, Utrecht, The Netherlands; 5 Institute of Infectious Disease and Molecular Medicine (IDM), University of Cape Town, Cape Town, South Africa

**Keywords:** Human microbiome, Global health equity, Translational ethics, Equitable partnerships, Regulatory science, Co-laboration

## Abstract

Human microbiome research is expanding globally, yet remains dominated by samples, institutions, and leadership from the Global North. This imbalance undermines scientific validity, as microbiomes are shaped by socio-ecological context and temporal dynamics, and risks producing diagnostics and therapeutics that are not applicable across diverse populations. In this comment, we engage with van Daele et al.’s framework of co-laboration and argue for ethical, interdisciplinary, and locally led research models that center community participation, context-rich metadata, and equitable authorship. We outline structural requirements—governance tools, funding mechanisms, and accountability systems—needed to ensure these frameworks are implemented and advance both scientific integrity and global health equity.

Video Abstract

**Supplementary Information:**

The online version contains supplementary material available at 10.1186/s40168-026-02371-3.

## Background

Human microbiome research is expanding globally, moving from largely descriptive to clinically motivated research with promise for diagnostics, therapeutics, and prevention across a range of diseases [1–3]. Yet, despite this expansion, the evidence base remains disproportionately skewed towards European and North American populations. A 2022 meta-study [4] reported that >70% of publicly available human microbiome samples originate from Europe, the USA, and Canada, while regions like South Asia and Sub-Saharan Africa, which comprise ~40% of the world’s population, contribute <10%. Similarly, a 2021 systematic review found that nearly 80% of publications reporting microbiome research conducted in Africa listed neither first nor last authors affiliated with African institutions [5]. These patterns highlight that the imbalance extends beyond sampling bias alone, reflecting deeper structural inequities in research agendas, funding flows, infrastructure, training, leadership opportunities, and authorship—inequities rooted in longstanding global power asymmetries [6].

The implications of this imbalance are far-reaching, influencing not only what is discovered but also how (and for whom) microbiome-based clinical applications are developed. Because microbiomes are strongly shaped by socio-ecological context [7, 8], study designs inattentive to place, time, and lived context risk generating knowledge that is neither generalizable nor clinically reliable. Temporal dynamics represent an additional, often overlooked dimension: sampling strategies that rely on single or infrequent time points implicitly assume microbial stability and may encode Global North norms of health, access to care, and biological regularity. Together, these assumptions increase the risk of misclassifying dynamic microbial states as pathology and of benchmarking diagnostics and therapeutics against reference ranges that do not translate across populations, particularly when strain-level functional diversity is treated as interchangeable, leading to reduced efficacy or unintended harm.

Beyond scientific validity, ethical harm arises when communities contribute biological samples without meaningful participation in agenda-setting, governance, or benefit-sharing. Inequitable collaborations further erode trust and perpetuate economic and academic dependency, leaving researchers in the less resourced settings under-recognized and structurally constrained in shaping future research priorities. These risks become most visible at the point of translation, where microbiome-informed diagnostics, live biotherapeutics, and preventive interventions are deployed beyond the populations in which they were developed. Here, ethical shortcomings upstream translate directly into regulatory uncertainty, variable efficacy, and inequitable health outcomes downstream.

## Main text

To address these intersecting challenges—(1) population and agenda bias, (2) limited community participation and benefit-sharing, and (3) inequitable leadership, authorship, and resource allocation—van Daele and colleagues propose the concepts of “co-laboration” and “co-laborative science” [9] to rebuild the field ethically and scientifically. Framed as a labor- and time-intensive, experimental mode of collaboration, co-laboration emphasizes radical interdisciplinarity and interculturality as prerequisites for ethical and scientifically robust microbiome research. By bringing together microbiologists, anthropologists, nutritionists, global health scholars, social scientists, and community representatives in sustained co-design and co-analysis, the authors seek to move beyond reductive binaries (such as “traditional vs. modern”), situate microbiomes within their socio-ecological realities, and co-create research trajectories that are locally meaningful while remaining globally informative. Importantly, this approach could be applied beyond research questions and data interpretation to the development of microbiome-based interventions themselves, including decisions around formulation, strain selection, dosing logic, and delivery context. Central to this framework is the recognition that trust, ownership, and fairness in the handling of data, samples, and downstream benefits must be embedded across the entire research lifecycle.

This is a compelling framework that treats ethics not as a regulatory afterthought, but as an integral research method. It aligns with recent editorial and policy shifts, including commitments by *Nature* and *The Lancet* to counter helicopter research, the Cape Town Statement on fairness and equity in research, and the explicit incorporation of health equity into the Declaration of Helsinki [10–13]. It also resonates with a growing body of scholarship calling for equitable and effective partnerships in microbiome research [14–17]. Importantly, recent large-scale initiatives demonstrating locally led sampling, context-rich metadata collection, and equitable authorship have shown that scientific excellence and research equity are not competing goals but mutually reinforcing ones [18–20].

The strength of van Daele *et al*.’s framework lies in its effort to translate critique into actionable guidance, particularly through its emphasis on long-term interdisciplinary and intercultural partnerships, shared agenda-setting, meaningful informed consent, and recognition of diverse knowledge systems. Nonetheless, key questions remain regarding operationalization. How will funding mechanisms be restructured to enable sustained local leadership? What equity benchmarks will be used to assess whether commitments translate into measurable change? In addition, as van Daele notes, researchers must be prepared to engage in genuinely symmetric co-laborations, yet this requirement demands deeper interrogation. Intersectional power dynamics—shaped by race, gender, nationality, class, and professional status—directly influence who is perceived as a credible leader and who feels permitted to fully participate. The positional authority afforded to individuals shapes not only how collaborators receive them, but also how they navigate their own participation. Attending to these layered inequities is essential if co-laboration is to be implemented. At the same time, it is important to recognize that frameworks like co-laboration are now often used as standard language in microbiome research, even when they lack real commitments. Without concrete structures, such as clear governance tools and accountability measures, these frameworks risk becoming little more than an empty consensus: widely referenced but rarely put into meaningful practice. In the end, they remain aspirational rather than truly transformative.

## Conclusions

To render co-laboration truly operational, research projects and consortia must move beyond statements of intent towards structural commitments. These include pre-registered authorship and leadership plans, ring-fenced budgets for community engagement and social science integration, investment in local infrastructure and sample repositories, equitable access to sequencing and secure data storage, reciprocal mobility between Global South and Global North partners to balance visibility and labor, and support for open-access dissemination. Such measures translate ethical principles into practice. However, because these mechanisms are rarely mandated, and some PIs do not view them as their responsibility, the principal barrier remains enforcement. In this sense, ethics in microbiome research is no longer primarily a conceptual challenge, but an institutional one. Unless funders, journals, and governments require and monitor these commitments, meaningful change is unlikely in the near term. For the sake of public health, and for the scientific integrity of the microbiome field, these commitments should be implemented now—not later.

## Data Availability

No datasets were generated or analysed during the current study.
